# Rationale, design and methodology of a double-blind, randomized, placebo-controlled study of escitalopram in prevention of Depression in Acute Coronary Syndrome (DECARD)

**DOI:** 10.1186/1745-6215-10-20

**Published:** 2009-04-07

**Authors:** Baiba Hedegaard Hansen, Jamal Abed Hanash, Alice Rasmussen, Jørgen Fischer Hansen, Morten Birket-Smith

**Affiliations:** 1 Liaison Psychiatry Unit, Psychiatric Centre Bispebjerg, Bispebjerg University Hospital, Bispebjerg Bakke 23, 2400 Copenhagen NV, Denmark; 2 Department of Cardiology, Bispebjerg University Hospital, Bispebjerg Bakke 23, 2400 Copenhagen NV, Denmark; 3 Department of Psychiatry, University Hospital of Copenhagen Rigshospitalet, Blegdamsvej 9, 2100 Copenhagen Ø, Denmark

## Abstract

**Background:**

The prevalence of depression in patients with acute coronary syndrome, i.e. myocardial infarction and unstable angina, is higher than in the general population. The prevalence of anxiety is higher as well. Both depression and anxiety are associated with poor cardiac outcomes and higher mortality. Comorbid depression in patients with acute coronary syndrome often goes undiagnosed, and it is therefore a challenging task to prevent this risk factor. The study of DEpression in Coronary ARtery Disease (DECARD) is designed to examine if it is possible to prevent depression in patients with acute coronary syndrome.

**Methods:**

Two hundred forty non-depressed patients with acute coronary syndrome are randomized to treatment with either escitalopram or placebo for 1 year. Psychiatric and cardiac assessment of patients is performed to evaluate the possibility of preventing depression. Diagnosis of depression and Hamilton Depression Scale are the primary outcome measures.

**Discussion:**

This is the first study of prevention of depression in patients after acute coronary syndrome with a selective serotonin reuptake inhibitor.

**Trial Registration:**

Identifier: NCT00140257

## Background

The prevalence of depression in patients recovering from acute coronary syndrome (ACS) defined as acute myocardial infarction (AMI) and unstable angina pectoris (UAP) has been reported to be 10–40% [1-5]. Depression after ACS is associated with increased mortality and morbidity [6]. Furthermore, cardiac patients with depression have an increased number of visits to general practitioners and are less likely to return to work [5]. Among patients who survive the first post-AMI year, depression is also associated with increased health care costs linked to both hospital readmissions and out-patient contacts [7]. The negative impact of depression has been observed not only in patients with an established diagnosis of depression, but also in patients who reported symptoms of depression during hospitalization [8].

Anxiety often occurs simultaneously with depression, and mixed anxiety-depression is found to be present in 90% of depressed patients after AMI [9]. After percutaneous coronary intervention (PCI) patients, who had symptoms of both anxiety and depression, reported poorer health status compared to patients, who only had anxiety or depression or no symptoms [10]. Furthermore, post-AMI anxiety is an independent predictor for both cardiac events and rehospitalizations [11,12]. A history of depression is associated with more frequent angina and poor quality of life after ACS [13]. Depression is related to severity of cardiac disease, depressive symptoms are more frequent in patients with left ventricle dysfunction [14] and have negative influence on the cardiovascular prognosis [6]. Only few randomized trials examined whether treating depression in cardiac patients could improve prognosis. ENRICHD (Enhancing Recovery in Coronary Heart Disease) included 2,481 post-AMI patients with major depression and (or) low social support. The study showed no effect of cognitive behavioural therapy on cardiac prognosis [15]. SADHART (Sertraline Antidepressant Heart Attack Randomized Trial) used the selective serotonin reuptake inhibitor (SSRI) sertraline for treatment of post-ACS depression. A reduction in depressive symptoms in patients treated with sertraline was found, but overall the impact on the score of Hamilton Depression Scale (HDS) was not significant [16]. In MIND-IT (Myocardial Infarction and Depression – Intervention Trial) study 331 depressed AMI patients were enrolled in double blind, placebo-controlled study with mirtazapine. No differences in depression status were found in patients in the mirtazapine arm of the study and the care-as-usual arm at 18 months post-AMI [17]. In Canada the CREATE (Cardiac Randomized Evaluation of Antidepressant and Psychotherapy Efficacy) study was conducted among 284 outpatients with coronary artery disease (CAD) and depression. The SSRI citalopram was found to be superior to placebo in reducing HDS scores, but no benefit of interpersonal psychotherapy was evident [18]. In a recent systematic review Thombs et al [19] identified 6 depression treatment trials in patients with cardiovascular disease. The treatment of depression was associated with modest improvement in depressive symptoms but no improvement in cardiac outcomes.

Several biological links have been proposed to explain the relationship between cardiac disease and depression. Heart rate variability (HRV) reflects the sympathetic/parasympathetic balance in the autonomic regulation of the heart, as well as the capacity of the autonomic nervous system to vary the intervals between consecutive heartbeats. HRV may be reduced in depressed patients with CAD [20-24], and it has been proposed, that treating depression in CAD patients with cognitive behavioural therapy may increase HRV [22]. Treatment with SSRIs appears to normalize low HRV in depressive patients without any heart disease [25], but it is still unknown whether it influences mortality.

Platelets play a crucial role in the development of atherosclerosis and ACS, and several studies have shown increased platelet activity in depressed patients with ischemic heart disease [26,27]. SSRIs influence platelet activity by blocking reuptake of serotonin and thereby reducing the activity of platelets. It has been demonstrated that SSRIs may even reduce platelet activity in cardiac patients without depression [28,29].

Vascular endothelial dysfunction also plays an important role in the relation between depression and heart disease. Arterial endothelial function measured by flow-mediated dilation is impaired in patients treated for depression [30,31] as well as in patients with documented coronary heart disease and depressive symptoms. Treatment with antidepressants seems to be associated with improved flow-mediated dilation [31].

The diagnosis of depression in post-ACS patients presents several problems in itself. In a significant number of these patients symptoms of depression are poorly recognized [32]. On the other hand, cardiac patients may be wrongly diagnosed as depressed due to diagnostic overlap of somatic symptoms such as fatigue and insomnia [1]. It is still unknown whether screening for depression is of benefit in patients with cardiovascular disease [19].

Based on the above mentioned studies we planned to test the hypothesis if 1-year treatment with a SSRI drug (escitalopram), which has beneficial cardiovascular effects, can prevent depression in post-ACS patients, and to examine the influence of the drug in cardiovascular variables known to be related to cardiovascular prognosis.

## Methods

### Overview

The DEpression in Coronary ARtery Disease (DECARD) study is an investigator-initiated and driven study and 240 non-depressed patients with ACS are enrolled. The patients enter a 1-year treatment, randomly assigned to either escitalopram or placebo.

### Eligibility criteria

#### Inclusion criteria

1. Admission to the coronary care unit for ACS [33];

2. Randomization within 8 weeks from index hospitalization for ACS;

3. Age > 18 years;

4. Women below 45 years of age may be enrolled in the study if they have a negative pregnancy test and use an effective mean of contraception;

5. Signed informed consent.

#### Exclusion criteria

1. Current depression based on Schedules for Clinical Assessment in Neuropsychiatry (SCAN) [34] diagnostic criteria of depression and/or a score of 13 or more on the 17-item HDS;

2. Use of antidepressants or antipsychotic medication within 4 weeks preceding enrolment;

3. Previous intolerance to SSRI;

4. Severe, life-threatening medical conditions implying, that the patient cannot participate in the 1-year study course;

5. Severe congestive heart failure defined as New York Heart Association (NYHA) functional class IV;

6. Current alcohol or substance abuse;

7. Psychosis or dementia;

8. Current participation in other intervention trials;

9. Pregnancy and lactation;

10. Linguistic difficulties or not mastering Danish.

### Recruiting Process

All patients admitted to two departments of cardiology at Bispebjerg and Amager University Hospitals, Copenhagen are screened for ACS. The diagnosis of ACS is made by the doctor in charge at the coronary care units. Patients, who meet the inclusion criteria and none of the exclusion criteria, are asked to participate in the study by a study doctor or nurse. Patients are recruited during their stay at the hospital or contacted after discharge within eight weeks from hospitalization.

In order to compare participants and non-participants, four questions from the Primary Care Evaluation of Mental Disorders (PRIME-MD) [35] screening questionnaire concerning mood and anxiety are administrated to all eligible patients. Patients are not in- or excluded on basis of the PRIME-MD screening.

**Figure 1:**
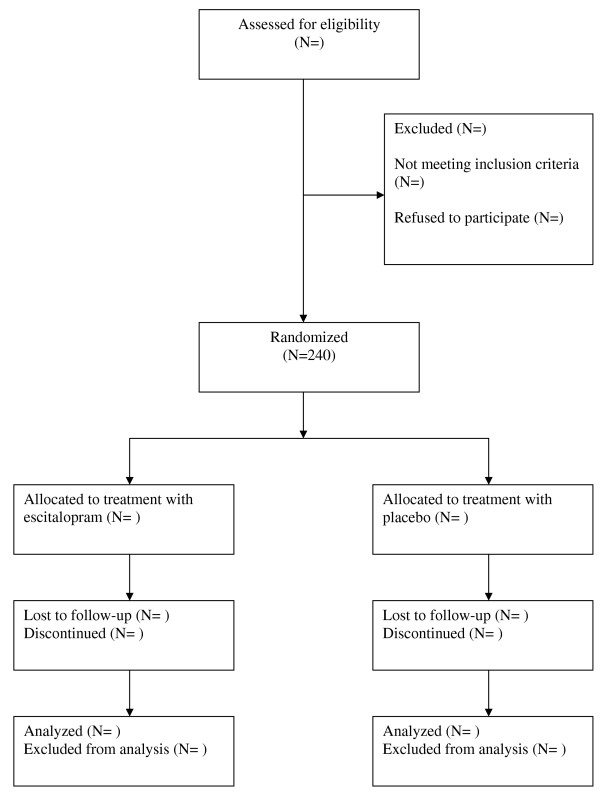
**flow chart**.

The potentially eligible patients are examined using SCAN interview. SCAN interview is applied by one of the three investigators in the study. (see Figure 1)

### Ethics and informed consent

The study is conducted in accordance with the Helsinki Declaration and is approved by The Danish National Committee on Biomedical Research Ethics, Identifier: (KF) 02-025/03, The Danish Medicines Agency, Identifier: 2612–2274, and The Danish Data Protection Agency, Identifier: 2004-54-1621.

All eligible patients receive written information and are simultaneously informed about the study by a study nurse. Before entering the study patients are informed about study objectives, study design and potential risks and benefits by one of the main investigators. Patients are also informed about their right to withdraw from the study at any time. Written consent is obtained from each patient. After informed consent the general practitioners are informed by mail about the patients entering in the study.

The DECARD trial is monitored by the Institute of Good Clinical Practice of University of Copenhagen.

### Randomization and blinding

After signing of the informed consent form, baseline data are collected and patients consecutively randomized to a double-blind treatment with either escitalopram or placebo. Patients are randomized in centrally prepared randomization blocs. The randomization is done independently of the researchers. The allocation sequence is implemented using the consecutive number of the study medication. Patients undergoing coronary artery bypass graft (CABG) surgery are stratified to a separate randomized group due to expected difference in outcome after major surgery versus PCI or non-invasive treatment.

Investigators, nurses, outcome assessors as well as patients remain blinded to group assignment during the entire intervention and data analysis.

### Psychiatric assessment

After demographic data collection during the baseline visit, SCAN is administrated using selected chapters regarding depression and anxiety (Part I, Chapters 3, 4, 6, 7, 8 and 21). This semi-structured diagnostic interview is used to exclude depression in patients entering the study. All investigators have completed the WHO certified training course in SCAN. Information on current and previous mental illnesses as well as the family history of mental illnesses is also obtained.

During all visits patients are assessed using the 17-items HDS [36], Hamilton Anxiety Scale (HAS) [37] and Clinical Global Impression (CGI) [38]. Tolerability of study treatment and possible side effects are registered by using the UKU Side Effect Rating Scale [39]. Inter-rater reliability is assured by regular HDS and HAS training sessions.

Furthermore, several self-administrated questionnaires are used for assessment of psychopathology. Beck Depression Inventory (BDI) [40] is administrated following the interview at every visit. At baseline and during fifth and ninth visit (i.e. weeks 0, 26 and 52) general psychopathology is assessed with the Symptom Checklist (SCL-92) [41], while function and social support is evaluated with the SF-36 Health Survey (SF-36) [42] and ENRICHD Social Support Instrument (ESSI) [43].

### Cardiac assessment

Cardiac evaluation is performed at baseline, weeks 26 and 52 at the end of treatment and includes information of risk factors for cardiovascular disease (smoking, diabetes, history of arterial hypertension, family history of CAD, physical activity), alcohol consumption, and current medications.

Canadian Cardiovascular Society (CCS) Classification for angina and NYHA functional class are estimated and arterial blood pressure measured.

A 24-hour Holter ECG monitoring is done at baseline and at weeks 26 and 52. All recordings are analysed at the ECG core laboratory at Hvidovre University Hospital to measure HRV and detect possible arrhythmias and silent myocardial ischemia.

A standard echocardiographic examination assessing left ventricular function is carried out at baseline and at weeks 26 and 52.

A 12-lead ECG is obtained to be analysed for atrioventricular and intraventricular conduction as well as QTc interval as cardiac safety measure.

Biochemical analysis includes full blood cell count, complete blood chemistry, fasting lipid profile, brain natriuretic peptide (pro-BNP), and High sensitivity-C-reactive protein (HS-CRP).

Patients, who due to depression or any other reason terminate before 52 weeks, are asked to complete final assessment equal to last visit in the study.

### Study medication

Study medication escitalopram (Cipralex) is provided by H. Lundbeck A/S, Denmark. Escitalopram is a highly selective SSRI with a fairly rapid onset of antidepressant activity. Escitalopram was chosen because its favourable side-effect profile and being a weak inhibitor of CYP2D6 it has a very low potential for drug-drug interactions [44].

The medication is packed by Pharmacy "Region Hovedstadens Apotek" and is delivered in identical blister packs for placebo and escitalopram. Medication is administered once daily. Code-break cards are stored in the Trial Master File in case the randomization code of the medication has to be broken before the end of the trial.

Patients receive an initial dose of 5 mg escitalopram or matching placebo (week 1) with dose increase to 10 mg (week 2). Although the dose of escitalopram used for treatment of depression is 20 mg we decided to use 10 mg as it is the recommended dose for people older than 65 years and a substantial part of study participants are older than 65 years. Pill count is performed at each visit.

Compliance is defined as taking at least 75% of study medication. In order to avoid disruption syndrome patients receive half dosage (5 mg escitalopram or placebo) for 14 days before the study medication is withdrawn.

### Intervention

Patients are seen at 9 visits during the year of study participation. Visits are spaced with two weeks between the first and second visit, 6 weeks between the second and the third visit and approximately 7 weeks between visits during the remaining study period. It is rendered important to see patients two weeks after initiation of treatment, in order to handle possible side effects of the study medication, insure compliance, and answer possible questions.

If patients during the study period develop HDS score 13 or more [45], the investigator increases the dose of escitalopram/placebo to 20 mg and patients are seen at an extraordinary visit approximately 14 days later, where the ICD-10 criteria for depressive episode are applied by a psychiatrist. Patients diagnosed as having a moderate or severe depressive episode, terminate the study medication and are offered an open treatment for depression.

Symptoms of anxiety are assessed at each visit using HAS, but the intervention is not changed or terminated in case of worsened symptoms of anxiety.

### Measurements of serotonin level in a subpopulation

In order to investigate the influence of long-term treatment with SSRI on serotonin level, a substudy is performed on 60 patients measuring the level of serotonin in platelets. A blood sample is drawn at baseline, after 26 weeks, 52 weeks as well as 4 weeks after discontinuation of study medication. This substudy is done in cooperation with Department of Clinical Biochemistry at Frederiksberg University Hospital.

### Endpoints

#### Primary endpoint

ICD-10 depressive episode (moderate, severe);

Hamilton Depression Scale score of 13 or more.

#### Secondary (safety) endpoints

1. changes in 24-hour and 15-minute day and night HRV (time- and frequency domains) over time and episodes ventricular arrhythmias registered by 24-hour ambulatory ECG;

2. changes in pro-BNP and HS-CRP over time;

3. incidence of cardiovascular events defined as recurrent ACS and unplanned revascularisation;

4. changes in self-rated physical health and quality of life (SF-36);

5. changes in anxiety level measured by HAS and SCL-92 anxiety scale;

6. utilization of health services during the study period;

### Sample size, data analysis and statistical methods

Sample size is calculated on the basis of the assumption, that 30% of patients the first year after ACS will develop a moderate or severe depressive episode [46] which can be reduced to 15% by SSRI treatment. Power calculation is based on a two-sided test, and with a power of 0.80 and the significance level α = 0.05, the required sample size in each group is 117. There are no interim efficacy analyses planned.

Data are entered in excel and then in SPSS. Data quality is assured by double-entering of 10% of all data. Data are analysed according to both intention-to-treat and per-protocol (efficacy population) principle using SPSS version 15 (SPSS Inc., Chicago, IL). Intention-to-treat population is defined as all randomized patients. Per-protocol analyses include patients taking study medication for more than 14 days and having no violation of eligibility criteria. The statistical analyses are performed blinded, i.e. analysed without breaking the code, and with the patients belonging to either group A or B.

The null hypothesis is that no difference will be demonstrated in the number of patients developing a moderate or severe depressive episode during the study period between the escitalopram and placebo groups. The cumulative incidences of outcomes (depressive episodes) as well as secondary outcomes (incidence of arrhythmias and cardiovascular events) by treatment group are examined by Kaplan-Meier plots. Subsequently, the relationship between the two treatments and depressive episodes is examined using the Cox proportional-hazards model with the study period as the underlying time. In subsequent models the relationship is adjusted for age, gender, social class, physical activity, psychiatric comorbidity and cardiac risk factors at baseline. A forward selection and a backward selection are conducted, excluding the non-significant variables for each model. Continuous variables are tested for linearity and if linearity is not present, the variables will be included as discrete variables in the models. The two treatment groups at baseline are compared using 2-way ANOVA for continuous measures and Chi-square tests for nominal data. Further analyses are two-factor mixed design, repeated measure with outcome variables after treatment as dependent variables and outcome variables before treatment as covariates. Missing data are imputed as last observation carried forward. The two treatment groups are between-subject factors and time 0, 6 and 12 months after treatment are within-subject factors. For each analysis a 95% confidence interval is derived. All tests of statistical significance are interpreted with a criterion of p < 0.05.

## Discussion

This paper reports the protocol of the first study recruiting non-depressed post-ACS patients for preventive treatment of depression with escitalopram or placebo. A negative impact of depression and anxiety on outcome of ACS is well-established, but depression and anxiety in ACS patients often remain unrecognized and untreated [2,9]. Most clinical trials concerning mental disorders in ACS have targeted the treatment of an already established depressive state. In contrast, we have designed a study of prevention in ACS patients definitely not having depression at baseline.

As depression worsen the prognosis of cardiac patients it might be possible to improve prognosis by prevention of depression using antidepressive medication. SSRIs can prevent recurrent depression [47] and depressive episodes in patients with diabetes [48].

Tricyclic antidepressants (TCAs) have been used with caution in ACS patients due to risk of serious adverse cardiac events. In contrast, SSRIs are a safe and effective medication in the cardiac population [16,49]. In a randomized study of the prevention of depression in stroke patients SSRI (sertraline) was safe and compared to placebo demonstrated a reduction in cardiovascular events [50].

Escitalopram is a SSRI developed to the treatment of patients with depression [44]. No long-term treatment studies with escitalopram in cardiac patients have yet been published, but a study with escitalopram assessing safety and tolerability of the drug in long-term treatment, i.e. 12 months in depressed patients, demonstrated that escitalopram is well-tolerated. The overall withdrawal rate was 26% and the withdrawal rate due to adverse events was 9%, which is lower than seen with most other antidepressants. Thus, escitalopram has a favourable adverse event profile [51].

The selection of SSRI in this trial is based on previous studies supporting their safety in patients with cardiovascular disease without any association with cardiovascular adverse events [52]. SSRIs block the reuptake of serotonin including in platelets, which contain but do not synthesize serotonin. Treatment with SSRIs leads to depletion of serotonin in platelets [53,54], this in turn results in lower activity of platelets [55] and reduced aggregation [56]. This supports the hypothesis that SSRIs may reduce the risk of myocardial infarction [57-59] as lower platelet serotonin content may lead to less serotonin release during platelet activation at an intra-coronary stenosis [60].

Bleeding might be a side effect of SSRI treatment, but studies have shown conflicting results.

Though several studies have suggested that SSRIs may increase the risk of bleeding [59,61-63], especially in patients also taking non-steroidal anti-inflammatory drugs (NSAIDs) [64], one study did not find an increased bleeding risk when NSAIDs and SSRIs were prescribed together, compared to taking the each drug separately [65]. Furthermore, treating depressed CAD patients with citalopram reduced HDS scores and showed no differences between citalopram and placebo in bleeding tendencies [18].

The cardioprotective properties of the SSRI's may also be explained by the SSRI's effect on heart rhythm. The SSRI sertraline in post AMI depressed patients facilitated the recovery of HRV, which is an expression of cardiac autonomic function [66].

Changes in cardiovascular risk factors may influence the long-term prognosis in post-ACS patients. By repeated registration of risk factors, HRV, and biochemical markers, i.e. serum lipids, CRP and pro-BNP, it is possible to describe differences and changes over time in cardiovascular risk in the two treatment groups and to evaluate interaction between risk factors and trial medication.

Several studies have examined other psychosocial risk factors than depression and several of these factors are included in the present study. Anxiety has been shown to be a cardiac risk factor [11], and is measured by a well known interview based instrument (HAS), as well as by self-rating (SCL-92). Social support and quality of life are measured with ESSI and SF-36, both instruments previously performing satisfactorily in studies on cardiac risk factors [43,67].

The clinical study was initiated at the end of 2004. Patients in this study are treated in two coronary care units in accordance with recent guidelines [68]. All patients with UAP and with post AMI signs of myocardial ischemia are referred for coronary angiography, and if indicated PCI or CABG. Post PCI patients with stent implantation are treated with clopidogrel for 12 months. Standard secondary prevention with beta-blockers, calcium channel antagonists, statins, ACE/angiotensin-II antagonists, and aspirin are used in accordance with the guidelines. All medication during the study period is monitored. The inclusion of patients was estimated to take 18 months but due to slower-than-expected enrolment was extended until the end of 2007. During the study period no significant changes in the guidelines for treatment of patients with ACS took place.

## Competing interests

The authors declare that they have no competing interests.

## Authors' contributions

All authors read and approved the final manuscript. BHA as an investigator in the study drafted the manuscript and has made a substantial contribution to acquisition of data. JH is responsible for the acquisition of cardiac data and he has revised manuscript critically. AR has designed the study and revised the manuscript. JFH has contributed to revising of manuscript for important intellectual content and he is a supervisor of the cardiologic part of the research project. MBS has designed the study, is supervisor of the research project and he has helped with revision of manuscript.
